# Study protocol: DexaDays-2, hydrocortisone for treatment of dexamethasone-induced neurobehavioral side effects in pediatric leukemia patients: a double-blind placebo controlled randomized intervention study with cross-over design

**DOI:** 10.1186/s12887-021-02896-6

**Published:** 2021-09-27

**Authors:** A. M. van Hulst, E. J. Verwaaijen, M. F. Fiocco, S. M. F. Pluijm, M. A. Grootenhuis, R. Pieters, E. L. T. van den Akker, M. M. van den Heuvel-Eibrink

**Affiliations:** 1 Princess Maxima Center, Heidelberglaan 25, 3584 CS Utrecht, The Netherlands; 2 Mathematical Institute Leiden University, Niels Bohrweg 1, 2333 CA Leiden, The Netherlands; 3 Erasmus MC- Sophia Children’s Hospital, Wytemaweg 80, 3015 CE Rotterdam, The Netherlands

**Keywords:** Acute Lymphoblastic Leukemia (ALL), Dexamethasone, Hydrocortisone, Neurobehavioral Side Effects, Behavior, Mood, Sleep, Health Related Quality of Life, Randomized Controlled Trial

## Abstract

**Background:**

Dexamethasone, a highly effective drug in treating pediatric acute lymphoblastic leukemia (ALL), can induce serious neurobehavioral side effects. These side effects are experienced by patients and parents as detrimental with respect to health related quality of life (HRQoL). Based on previous studies, it has been suggested that neurobehavioral side effects are associated to cortisol depletion of the mineralocorticoid receptor in the brain. Our previously reported randomized controlled trial, the Dexadagen study (NTR3280), suggests that physiological hydrocortisone addition during dexamethasone treatment may overcome clinically relevant neurobehavioral problems in patients who experience these problems during dexamethasone treatment. With our current study, we aim to replicate these results in a targeted larger sample before further implementing this intervention into standard of care.

**Methods:**

In a national center setting, pediatric ALL patients between 3 and 18 years are enrolled in an Identification study, which identifies patients with clinically relevant dexamethasone-induced neurobehavioral side effects using the Strengths and Difficulties Questionnaire (SDQ). Contributing factors, such as genetic susceptibility, dexamethasone pharmacokinetics as well as psychosocial and family factors are studied to determine their influence in the inter-patient variability for developing dexamethasone-induced neurobehavioral side effects.

Patients with clinically relevant problems (i.e. a rise of ≥ 5 points on the SDQ Total Difficulties Score after 5 days of dexamethasone) are subsequently included in a randomized double-blind placebo-controlled trial with a cross-over design. They receive two courses placebo followed by two courses hydrocortisone during dexamethasone treatment, or vice versa, each time at least 16 days without study medication in between. The primary endpoint is change in SDQ score. The secondary endpoints are sleep (measured with actigraphy and the Sleep Disturbance Scale for Children) and HRQoL (Pediatric Quality of Life Questionnaire).

**Discussion:**

The results of our current study may contribute to the management of future ALL patients who experience dexamethasone-induced neuropsychological problems as it may improve HRQoL for patients who suffer most from dexamethasone-induced neurobehavioral side effects. Furthermore, by investigating multiple risk factors that could be related to inter-patient variability in developing these side effects, we might be able to identify and treat patients who are at risk earlier during treatment.

**Trial registration:**

Medical Ethical Committee approval number: NL62388.078.17. Affiliation: Erasmus Medical Centre. Netherlands Trial Register: NL6507 (NTR6695). Registered 5 September 2017

## Background

Dexamethasone, a highly effective drug for the treatment of pediatric acute lymphoblastic leukemia (ALL) [1–3], can induce serious neurobehavioral side effects. These side effects are experienced as particularly detrimental to health-related quality of life (HRQoL) by patients and parents [4]. Recent studies emphasize that the mineralocorticoid receptor (MR) in the brain plays an important role in the regulation of mood, behavior and sleep [5, 6]. Both the glucocorticoid receptor (GR) and MR are important for the binding of endo- and exogenous glucocorticoids [5]. In animals as well as humans it has been shown that the MR plays an important role in behavior, cognition and psychiatric diseases [6–11]. Besides MR expression in the brain, cortisol affinity and MR:GR balance are thought to be associated with behavior. It has been shown, that the MR has a tenfold greater affinity for endogenous cortisol than the GR [12]. Synthetic glucocorticoids mostly have the GR as their therapeutic target: dexamethasone has a high potency to activate GRs, but does not bind MRs [13]. In patients treated with glucocorticoids the production of endogenous cortisol is suppressed. Therefore, in patients treated with high doses dexamethasone, the hypothesis is that the GR in the brain is stimulated, whereas the MR is underactivated. The disturbance of this GR:MR balance conceivably deregulates the stress-system and enhances vulnerability to stress-related problems [5].

Consequently, we previously hypothesized that pediatric ALL patients who receive dexamethasone treatment, cortisol depletion of the MR in the brain may be responsible for attendant neurobehavioral problems. We therefore performed a randomized controlled trial (RCT), the DexaDays-1 trial, to investigate whether these side effects could be ameliorated by adding a physiological dose of hydrocortisone which stimulates the MRs in the brain in a physiological way [14]. No beneficial effect of hydrocortisone on neurobehavioral problems could be shown in the complete group of 46 patients. However, in a small subgroup of patients with clinically relevant dexamethasone-induced neurobehavioral or sleeping problems (n = 16 and n = 9 respectively), hydrocortisone addition had a significant beneficial effect [14]. Our results suggest that neurobehavioral and sleeping problems can be reduced in children who are most affected. Before implementing this into standard clinical practice, we felt that the results require replication in a larger patient cohort with clinically relevant dexamethasone-induced neurobehavioral problems. Hence, we initiated the DexaDays-2 trial in 2018.

Several factors may be associated to neurobehavioral side effects during dexamethasone treatment which warrant further study.

Firstly, the role of genetic variation is evaluated. Several studies found single nucleotide polymorphisms (SNPs) in the MR and GR gene, which could contribute to inter-individual differences in increased glucocorticoid sensitivity and neurobehavioral and sleeping problems[7, 9, 12, 15–20]. Carrier status of specific relevant SNPs which have been linked before to psychopathology or sleeping problems may be associated to dexamethasone-induced side effects.

Secondly, dexamethasone pharmacokinetics may play a role. Dexamethasone clearance is higher in younger children, hence taking an inter-patient variability in dexamethasone levels during maintenance phase into account is important [21].

Thirdly, psychosocial and environmental factors may influence the severity of neurobehavioral side effects. It has been previously shown that the child’s distress during procedures in childhood cancer treatment is associated with parental distress [22]. Parental stress is associated with behavioral problems in children[23]. Overall, parental stress could potentially accelerate the development of dexamethasone-induced behavioral problems [24]. Furthermore, some social (family) risk factors, but also psychosocial support can influence coping strategies of parents and may thereby influence their perceptions of the problems caused by dexamethasone [25, 26].

## Methods

### General study design

**Fig. 1:**
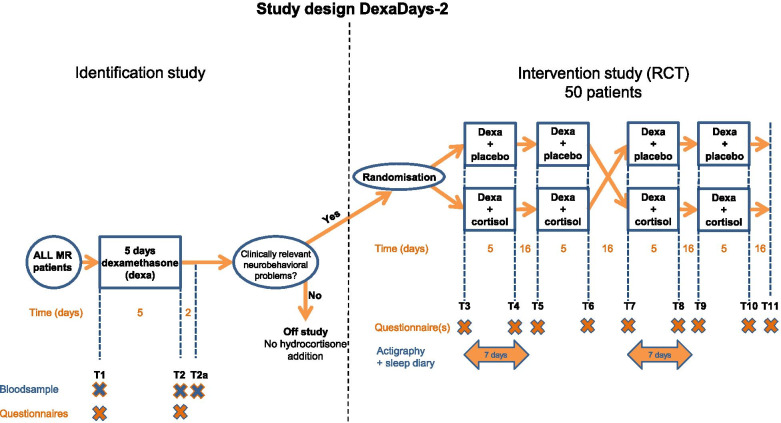
Study Design DexaDays-2. Schematic study design of the DexaDagen-2 study. See Table 1 (Identification study) and Table 2 (RCT) for the specification of blood samples and questionnaires. RCT = randomized controlled trial. ALL = acute lymphoblastic leukemia. MR = medium risk. Dexa = dexamethasone

The DexaDays-2 study is a Dutch national study and is coordinated from the Princess Máxima Center for pediatric oncology. The study consists of two parts: an *Identification study* (T1-T2) and a *Randomized Controlled Trial (RCT)* (T3-T11)*.* Figure 1 gives a schematic overview of the complete study. Tables 1 and 2 depict the content of all measurements in the Identification study and RCT respectively.

**Table 1:** Procedures in the Identification study

**Day **	**1**	**2**	**3**	**4**	**5**	**6**	**7**	**8**
**Timepoint Procedure**	**T1**					**T2**		**T2a**
Standard medication								
* Dexamethasone*	X	X	X	X	X			
* Vincristine*	X							
* Methotrexate*	X							X
Questionnaires								
* SDQ*	X					X		
* SDSC*	X					X		
* PSI*	X					X		
* DT*	X							
* DT (short)*						X		
* Support*	X							
* Support (short)*						X		
* Eating thermometer*	X					X		
Blood sample								
* Genetics*	X							
* Dexa peak level*	X							
* Dexa trough level*						X		X
Somatic parameters								
* Weight*	X					X		X
* Height*	X					X		X
* Blood pressure*	X					X		X
Done at (h = home / H = hospital)	H	h	h	h	h	H	h	H

The Identification study is the cohort from which eligible candidates for the Randomized Controlled Trial are selected. T1 = start dexamethasone. T2 = after 5 days of dexamethasone. T2a = 2 days after stop dexamethasone. See Fig. 1 for a schematic overview

*SDQ*  Strengths and difficulties questionnaire, *SDSC* Sleep disturbance scale for children, *PSI*  Parenting stress index, *DT*  Distress thermometer, *PedsQL* Pediatric quality of life questionnaire

**Table 2:** Procedures and intervention in the RCT

Day	1 / 43	2 / 44	3 / 45	4 / 46	5 / 47	6 / 48	7 / 49	22 / 64	23 /	24 /	25 /	26 /	27 / 69	85
Timepoint Procedure	T3/T7					T4/T8		T5/T9	65	66	67	68	T6/T10	T11
Standard medication														
* Dexamethasone*	X	X	X	X	X			X	X	X	X	X		X
* Vincristine*	X							X						X
* Methotrexate*	X							X						X
Study medication														
*Hydrocortisone or placebo*	X	X	X	X	X			X	X	X	X	X		
Questionnaires														
* SDQ*	X					X		X					X	X
* SDSC*	X					X								
* PedsQL generic*	X					X								
* DT (short)*	X					X								
* Eating thermometer*	X					X								
Sleep diary	X	X	X	X	X	X	X							
Actigraphy	X	X	X	X	X	X	X							
Somatic parameters														
* Weight*	X					X		X						X
* Height*	X					X		X						X
* Blood pressure*	X							X						X
Done at (h = home / H = hospital)	H	h	h	h	h	h	h	H	h	h	h	h	h	H

T3/T7 & T5/T9 = start of dexamethasone and study medication. T4/T8 & T6/T10 = after 5 days of dexamethasone and study medication. T11 = closing visit. See Fig. 1 for a schematic overview

*RCT* Randomized controlled trial, *SDQ* Strengths and difficulties questionnaire, *SDSC* Sleep disturbance scale for children, *PedsQL* Pediatric quality of life questionnaire, *DT* Distress thermometer

### In- and exclusion criteria

Every Dutch ALL patient is screened on in- and exclusion criteria. After permission of their pediatric oncologist, eligible patients are approached by the study team. Patients are eligible if they fulfill the following criteria: age 3–18, confirmed diagnosis of acute lymphoblastic leukemia (ALL), inclusion in DCOG ALL MRG protocol and able to comply with scheduled follow-up. Only patients between 3 and 18 years can participate because our questionnaires are validated for these ages. Exclusion criteria are: patient or parent refusal, anticipated compliance problems, underlying conditions which affect the absorption of oral medication, pregnant or lactating patients, current uncontrolled infection or any other complications which may interfere with dexamethasone treatment, language barrier, pre-existing mental retardation, current hydrocortisone use or risperidone use.

In addition, to be eligible for the RCT, a patient has to show a rise of five or more points on the SDQ Total Difficulties scale after five days of dexamethasone treatment.

### Randomized Controlled Trial

The main study is a prospective double-blind placebo-controlled randomized trial (RCT) with a cross-over design. The primary aim of the RCT is to replicate the finding that addition of physiological doses of hydrocortisone to standard dexamethasone treatment reduces neurobehavioral side effects in pediatric ALL patients who suffer from clinically relevant dexamethasone-induced neurobehavioral problems. Neurobehavioral problems are measured with the parent-reported Strengths and Difficulties Questionnaire in Dutch (SDQ) [27] at every time point (T3-T11) (Fig. 1 and Table 2).

The secondary aim is to estimate the percentage of patients with clinically relevant dexamethasone-induced sleeping problems and replicate our previous finding that addition of physiological doses of hydrocortisone to standard dexamethasone treatment reduces these sleeping problems. Sleeping difficulties are measured using the Sleep Disturbance Scale for Children (SDSC) [28, 29] at T3 + 4 and T7 + 8, i.e. before and after one course hydrocortisone and one course placebo. Sleep is measured through actigraphy as well [30]. Patients wear a nonintrusive wrist actigraph for seven days [31]. Parents are asked to keep a sleep log during the actigraphy measurement to interpret the data. These measurements take place twice: once when a patient receives hydrocortisone and once during placebo.

We also evaluate whether hydrocortisone addition improves HRQoL in patients with dexamethasone-induced clinically relevant neurobehavioral problems. HRQoL is measured with the Pediatric Quality of Life questionnaire (PedsQL)[32]. The PedsQL is filled in before and after a hydrocortisone and placebo course, at T3 + 4 and T7 + 8.

#### Randomization and blinding

Patients are allocated to start with hydrocortisone or placebo using the method of a prefixed randomization list. This randomization list is prepared by the pharmacy, independent of the clinical investigators. The study is double blinded. Blinding of subject, researchers and physicians is ensured through use of the investigational medicinal product (IMP) and an identical placebo solution. In case of problems regarding study medication, the randomization list is available 24 h per day through the pharmacy.

#### Investigational treatment

The IMP is hydrocortisone solution, given orally. The drug is administered in physiological dosages of 10 mg/m2/day. Patients use hydrocortisone (1 mg/ml) or placebo three times daily divided in a ratio of 5:3:2, following the physiological circadian rhythm.

Patients receive hydrocortisone (two consecutive courses) or placebo (two consecutive courses) in a randomized order during a five-day dexamethasone treatment. A washout period of at least 2 weeks and 2 days is always present between courses to prevent carry-over effect. After 2 courses cross-over takes place (Fig. 1). The washout period renders the carry-over effect in the next period negligible. The idea is to use each patient as his own control by trying both regiments at different times and comparing the results.

Pharmacovigilance is guaranteed by measuring the fluid volume in the medicine bottles after each 5-day course of study medication.

#### Power calculation for the primary outcome parameter

A sample size of 23 pairs with a correlation equal to 0 achieves 79% power to detect a difference. of -5,2 between the null hypothesis mean difference of 0 and the actual mean difference of -5,2 at the 5% significance level (alpha) using a two-sided Wilcoxon Signed-Rank Test. These results are based on 3000 Monte Carlo samples from the null distribution: Normal with mean 3.4 and standard deviation 5.4 and the alternative distribution Normal with mean 8.6 and standard deviation equal to 6.3. Power computations are performed with PASS 2020 Power Analysis & Sample Size (https://www.ncss.com/software/pass/). We will include 50 patients in our RCT.

### Identification study

**Fig. 2:**
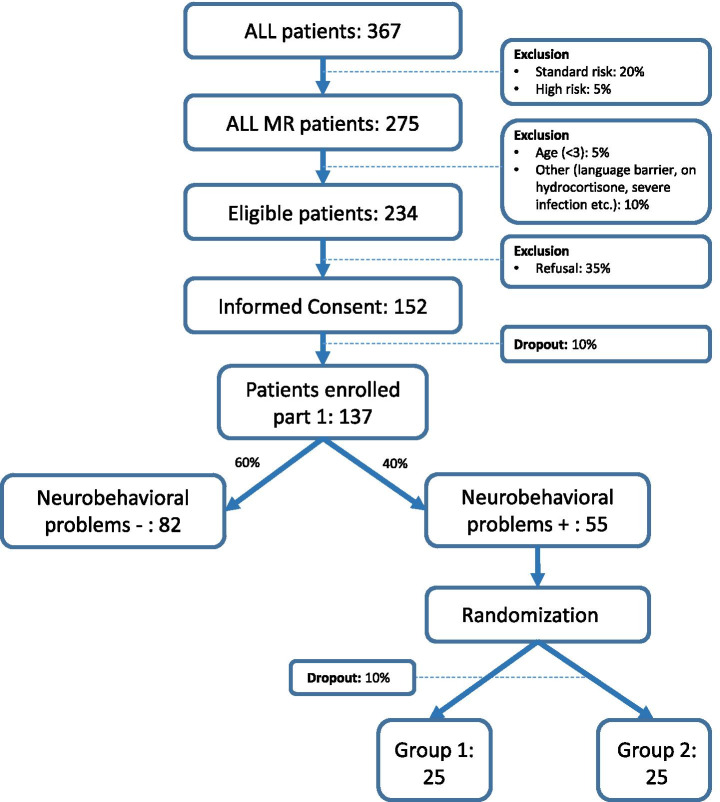
Flow chart. Expected number of ALL patients in the DexaDays-2 study. ALL = acute lymphoblastic leukaemia. Group 1: starting with two courses hydrocortisone, thereafter cross over to placebo. Group 2: starting with two courses placebo, thereafter cross over to hydrocortisone

The Identification study aims to select eligible patients for the RCT. Based on our previous study, we estimate that 40% of the included ALL patients experience clinically relevant neurobehavioral side effects [14]. Estimating the probability of a 10% dropout rate, a 35% refusal rate and exclusion of 15% based on our exclusion criteria, a total of approximately 150 patients will be included in the Identification study course (Fig. 2).

The secondary aims for studies in the Identification cohort are to investigate possible factors associated to the inter-patient variability in dexamethasone-induced neurobehavioral problems, including pharmacokinetics, candidate single nucleotide polymorphisms (SNP) analyses and psychosocial and environmental factors. Patients with a rise of five or more points on the SDQ Total Difficulties score after five days of dexamethasone (T1-T2, Fig. 1) will be compared with patients with a rise of four or less points. Dexamethasone kinetics are measured through peak levels (measured 2–3 h after the first dexamethasone administration on day 1 of the dexamethasone course (T1)) and trough levels (measured on day 6 (T2), after the last dexamethasone dose the previous evening). To identify possible very slow metabolizers, an additional blood sample will be taken on day 8, i.e. two full days after the last dexamethasone dose (T2a). A blood sample to evaluate carrier status of several relevant candidate SNP is taken on T1. Germline DNA will be extracted and candidate SNP analysis of the GR gene (NR3C1), including Bcl1 polymorphism (rs41423247 variant), ER22/23EK polymorphism (rs6189) and N363S/A1220G polymorphism, and the AHSG gene (rs4918 variant) will be included. Psychosocial and environmental factors include parenting stress, measured with the NOSI-K (Nijmeegse Ouderlijke Stress Index [33]), i.e. the adapted and shortened Dutch version of the Parental Stress Index (PSI)[34] and the Distress Thermometer (DT)[35, 36].

Several questions about received psychosocial support and education are filled in at T1 and T2. Eating and hunger satiety are measured using an Eating thermometer (a visual analogue scale to indicate hunger).

### Statistical analysis

The effect of treatment (*n* = 50) is assessed by comparing placebo with hydrocortisone on SDQ Total Difficulties Score (delta scores; subtracting the score on treatment day 1 from the score after treatment day 5) by employing a Paired Student’s T-test or Wilcoxon Signed Rank test in case of violation of normality assumption. SDQ sub scores will also be compared between the two groups. The effect of hydrocortisone on sleep and HRQoL (total and sub scores) is evaluated in the same way.

Due to the presence of repeated measures in the design of the RCT a generalized mixed model will be estimated to study the effect of therapy on neurobehavioral outcomes. This model explicitly accounts for the correlations between repeated measurements within each patient. Results from this analysis will provide information about the longitudinal effect of the treatment. A treatment period interaction will be included in the model to investigate the groups effect over time.

To study the associations in the Identification group between potential determinants (genetics, pharmacokinetics and environmental factors) and the occurrence of dexamethasone-induced neurobehavioral problems a binary logistic regression model will be estimated. Odds ratios along with 95% confidence interval will be provided.

### Data, monitoring and publication

All data is collected and stored in agreement with good clinical practice (GCP) guidelines. Certified members of the study team collect data on paper case report forms. OpenClinica Enterprise Version 3.13 is used to further collect and manage data. Blood is stored for 15 years. Deblinding takes place at the end of the study, after which the database will be frozen.

All questionnaires are web based and data is collected through a secure website, www.hetklikt.nu, a safe internet environment, which is widely used in pediatric (oncology) care in the Netherlands [37].

Adverse events are recorded, and all serious adverse events are reported to the competent authority by the investigator without undue delay, according to GCP. Patients can discontinue study participation at all times, without providing a reason for withdrawal. Standard insurance contracts apply in case of any unforeseen harm. Since patients are treated for a short time frame (2 × 5 days) and the drug under investigation is well characterized and given in a physiological dose, we do not expect any suspected unsuspected serious adverse reactions [14].

An independent certified third party (Julius Clinical) monitors the study. All processes including informed consent procedure, data collection and data management are monitored by this party. Monitoring takes place twice per year.

The results of this study will be disclosed unreservedly in the form of scientific publications. Participants are notified of study proceedings through regular newsletters.

When necessary the protocol can be modified or additions can be made. This can be done through amendments, which need approval by the Medical Ethical Committee.

## Discussion

This paper describes the DexaDays-2 protocol: a randomized controlled trial set up to replicate the finding that addition of physiological doses of hydrocortisone to standard dexamethasone treatment reduces neurobehavioral side effects in pediatric ALL patients. Our previous study suggests that patients with clinically relevant neurobehavioral problems benefit from treatment with hydrocortisone [14]. Currently no other satisfying options to treat dexamethasone-induced neurobehavioral problems are available [38]. The results of this study may affect the management of future ALL patients with dexamethasone-induced side effects as it may improve HRQoL for those who suffer most from these problems. Our study could also be important for adult patients or children with other conditions who receive dexamethasone and experience the accompanying neurobehavioral side effects [38, 39]. Furthermore, by investigating possible risk factors that could influence the inter-patient variability, we might be able to identify patients at risk for dexamethasone-induced neurobehavioral problems at an earlier stage, providing a possible intervention. Besides earlier recognition, the potential identification of risk factors for dexamethasone-induced neurobehavioral problems might lead to new outcomes which could be targeted to deal with these problems. For example, parenting stress or received support could be established as risk factors, providing starting points for non-pharmacological interventions.

Several strong points of this study can be discussed. First, every patient with ALL in the Netherlands can be screened on eligibility for this study, rendering a large and hopefully unbiased population. From this population, we select patients who might benefit from the intervention, following the results of our previous DexaDagen-1 study. Second, the design, a double-blind placebo controlled randomized controlled trial with cross-over, will minimize the risk of bias in our RCT. Third, we measure the effect of hydrocortisone in two subsequent dexamethasone courses. This is an addition to our previous study protocol, by which we want to mimic the normal situation of repetitive dexamethasone courses, and to investigate whether the possible effect of hydrocortisone is lasting.

Some possible study limitations have to be taken into account as well. To begin with, some patients might already use hydrocortisone therapy because patients heard of positive results from participants in our previous DexaDays-1 study. We address this problem by communicating the importance of our current study with all treating pediatric oncologists and asking them to include these patients in the study. Furthermore, because patients are coming to the Princess Máxima Center from the whole country, one extra visit might be a barrier for patients living far away. We try to overcome this problem by offering reimbursement of travel expenses and making it possible to visit a patient at home if the extra visit is the only objection of parents to participate. Another potential limitation is that mainly patients who experience dexamethasone-induced neurobehavioral problems are motivated to participate in the trial. This could result in too few children without neurobehavioral problems and this may affect the study of possible determinants of dexamethasone-induced side effects. To investigate the presence of possible bias, we ask non-responders to fill in a short non-obligatory questionnaire with questions regarding dexamethasone-induced side effects. This allows for comparison between participants and non-participants. If too few patients are included in the Identification study when we reach 50 patients in the RCT, it may be possible to continue the Identification study to generate a larger group to answer our secondary research questions by amending the protocol.

In conclusion, this study is set up to establish whether hydrocortisone addition to standard dexamethasone treatment is an effective therapy for dexamethasone-induced serious neurobehavioral side effects. With this therapy we aim to improve health related quality of life for ALL patients who suffer from this side effect during their 1.5 year treatment schedule.

## Data Availability

The data collection is ongoing. When the dataset is completed and results are published, it will be available from the corresponding author upon reasonable request.

## Abbreviations

ALL: Acute lymphoblastic leukemia
DCOG: Dutch Childhood Oncology Group
GCP: Good clinical practice
GR: Glucocorticoid receptor
HRQoL: Health related quality of life
MR: Mineralocorticoid receptor
MRG: Medium Risk Group
RCT: Randomized controlled trial
